# Theoretical Investigations of Si-Ge Alloys in *P*4_2_/*ncm* Phase: First-Principles Calculations

**DOI:** 10.3390/ma10060599

**Published:** 2017-05-31

**Authors:** Zhenyang Ma, Xuhong Liu, Xinhai Yu, Chunlei Shi, Fang Yan

**Affiliations:** Tianjin Key Laboratory for Civil Aircraft Airworthiness and Maintenance, Civil Aviation University of China, Tianjin 300300, China; hxliu@126.com (X.L.); xhyucauc@126.com (X.Y.); clshi01@126.com (C.S.); fyancauc@126.com (F.Y.)

**Keywords:** Si-Ge alloys, mechanical properties, anisotropic properties, electronic properties, 71.15.Mb, 71.20.-b, 62.20.de

## Abstract

The structural, mechanical, anisotropic, electronic and thermal properties of Si, Si_0.667_Ge_0.333_, Si_0.333_Ge_0.667_ and Ge in *P*4_2_/*ncm* phase are investigated in this work. The calculations have been performed with an ultra-soft pseudopotential by using the generalized gradient approximation and local density approximation in the framework of density functional theory. The achieved results for the lattice constants and band gaps of *P*4_2_/*ncm*-Si and *P*4_2_/*ncm*-Ge in this research have good accordance with other results. The calculated elastic constants and elastic moduli of the Si, Si_0.667_Ge_0.333_, Si_0.333_Ge_0.667_ and Ge in *P*4_2_/*ncm* phase are better than that of the Si, Si_0.667_Ge_0.333_, Si_0.333_Ge_0.667_ and Ge in *P*4_2_/*mnm* phase. The Si, Si_0.667_Ge_0.333_, Si_0.333_Ge_0.667_ and Ge in *P*4_2_/*ncm* phase exhibit varying degrees of mechanical anisotropic properties in Poisson’s ratio, shear modulus, Young’s modulus, and universal anisotropic index. The band structures of the Si, Si_0.667_Ge_0.333_, Si_0.333_Ge_0.667_ and Ge in *P*4_2_/*ncm* phase show that they are all indirect band gap semiconductors with band gap of 1.46 eV, 1.25 eV, 1.36 eV and 1.00 eV, respectively. In addition, we also found that the minimum thermal conductivity *κ*_min_ of the Si, Si_0.667_Ge_0.333_, Si_0.333_Ge_0.667_ and Ge in *P*4_2_/*ncm* phase exhibit different degrees of anisotropic properties in (001), (010), (100) and ($0\bar{1}0$) planes.

## 1. Introduction

As the most widely used semiconductor material, diamond silicon has many advantages: silicon is abundant in the earth, so the manufacturing cost is lower than that of other semiconductor materials; the diamond silicon purification process has been developed for several decades and has reached the highest level of human beings; there is extensive research on the doping and diffusion of silicon, and much experience has been gained. However, the electron and the hole mobility of diamond silicon is lower than that of other semiconductor materials, so it is difficult to meet the demand of higher performance semiconductor devices in the future. Diamond silicon belongs to indirect band-gap semiconductor materials, as light emission efficiency is not high enough. The inventions of the first transistor and the first IC (integrated circuit) based on the diamond germanium brung us two Nobel Prizes. The hole mobility of diamond germanium is four times that of diamond silicon and the electron mobility is about two times that of diamond silicon; the band gap of diamond germanium is relatively small, which is beneficial to the development of low voltage devices and the reduction of current leakage. However, diamond germanium belongs to active material. The defect of GeO is generated because redox reaction occurs easily on the dielectric material interface, along with other defects, so the performance of diamond germanium is significantly affected. In addition, the GeOI (germanium-on-insulator) technology is necessary for the development of future devices because diamond germanium cannot be directly used as substrate. Though this technology has some difficulties, it is generally believed that these difficulties will be overcome by researching diamond silicon material in the near future. With the goal of improving the performance of semiconductor devices, reducing power consumption and designing advanced microelectronic applications, diamond silicon is no longer able to meet the demand.

In order to solve this problem, more and more new phases of silicon and germanium with direct band gaps and better light absorption are studied [1,2,3,4,5,6,7,8,9,10]. In addition, the theme of new phases of Si-Ge alloys research [11,12,13,14,15] is of significant importance since new types of Si-Ge crystals are keys to the next generation devices. Si_1−*x*_Ge*_x_* alloys have been studied a lot in recent years due to their applications in both the optoelectronics and microelectronics industry. Zhang et al. [12] investigated the structural stability, dynamical, elastic and thermodynamic properties of Si–Ge, Si–Sn and Ge–Sn alloys in zinc blende structure by using first-principles calculations. The calculated cohesive energies and formation energies indicate that Si–Ge has the highest structural stability and Ge–Sn has the strongest alloying ability. Zhu et al. [13] investigated the structure, formation energy, and thermodynamic properties of Si_0.5_Ge_0.5_ alloys in zinc blende phase and rhombohedra phase through first-principles calculations. Bautista-Hernandez et al. [16] found a stable structure of silicon and germanium in the monoclinic (M phase) and orthorhombic structures (Z phase), respectively. From these works, both the M and Z phases happen to be mechanically and dynamically stable and the energy of these two phases for Si and Ge are slightly larger than that of Si and Ge in diamond structure. Therefore, these phases can be synthetized at room temperature. Using first-principles calculations, a new orthorhombic structure (Z-phase) property of Si_0.5_Ge_0.5_ alloy is carried out by Zhang et al. [17]. However, the Si_0.5_Ge_0.5_ alloy in the Z-phase is also an indirect semiconductor. A systematic density functional theory calculation has been performed on Si_12_, Ge_12_ and Si–Ge alloys in *P*4_2_/*mnm* phase by Fan et al. [18], including the stability, mechanical properties, anisotropic properties and electronic properties. The electronic structure calculations using the alloys are calculated by Heyd–Scuseria–Ernzerhof (HSE06) hybrid functionals, and reveal that Si_12_ in *P*4_2_/*mnm* phase is a semiconductor with an indirect band gap of 1.24 eV, Ge_12_ in *P*4_2_/*mnm* structure is a direct band gap semiconductor with the band gap of 0.71 eV, and Si_8_Ge_4_ and Si_4_Ge_8_ are indirect band gap semiconductors with the band gap of 1.25 and 1.03 eV, respectively.

This paper presents two new Si-Ge alloys in *P*4_2_/*ncm* phase; their physical properties, such as structural, mechanical, elastic, electronic and thermal properties are investigated by using first-principles calculations. The original structures of silicon and germanium in *P*4_2_/*ncm* phase were reported in Ref. [19].

## 2. Materials and Methods

The computational investigations were performed under the framework of density functional theory (DFT) [20,21] as implemented in the Cambridge Serial Total Energy Package (CASTEP) code [22]. The Kohn–Sham equations are solved within the framework of DFT by expanding the wave functions of valence electrons in a basis set of plane waves with kinetic energy smaller than a specified cut-off energy, *E*cut. Plane wave cut-off energy *E*cut of 340 eV and 2π × 0.025 Å^−1^ (8 × 8 × 4/8 × 8 × 4/7 × 7 × 4) grid of Monkhorst–Pack [23] points have been employed for Si, Si_0.667_Ge_0.333_, Si_0.333_Ge_0.667_ alloys in *P*4_2_/*ncm* phase and *E*cut = 300 eV and high Monkhorst–Pack points 2π × 0.025 Å^−1^ (7 × 7 × 4) have been employed for Ge in *P*4_2_/*ncm* phase to ensure well convergence of the computed structures and energies in this work. The presence of tightly-bound core electrons was represented by ultra-soft pseudo-potentials [24]. The exchange-correlation potential is treated within the local density approximation (LDA), developed by Ceperly and Alder, and parameterized by Perdew and Zunger (CA-PZ) [25,26] and Perdew–Burke–Ernzerhof (PBE) [27], developed by generalized gradient approximation (GGA). The structural parameters were determined using the Broyden–Fletcher–Goldfarb–Shenno (BFGS) algorithm [28], with the flowing thresholds for converged structures: energy change less than 5 × 10^−6^ eV per atom, residual force below 0.01 eV/Å, stress less than 0.02 GPa and the displacement of atoms during the geometry optimization less than 0.0005 Å. The phonon frequencies were calculated using linear response theory [29]. Other works [30,31] also give more credibility of the whole concept applied to diversified binary systems. The electronic band structures of the Si, Si_0.667_Ge_0.333_, Si_0.333_Ge_0.667_ and Ge in *P*4_2_/*ncm* phase were calculated utilizing the Heyd–Scuseria–Ernzerhof (HSE06) [32,33] hybrid functional.

## 3. Results and Discussion

### 3.1. Structural Properties

**Figure 1:**
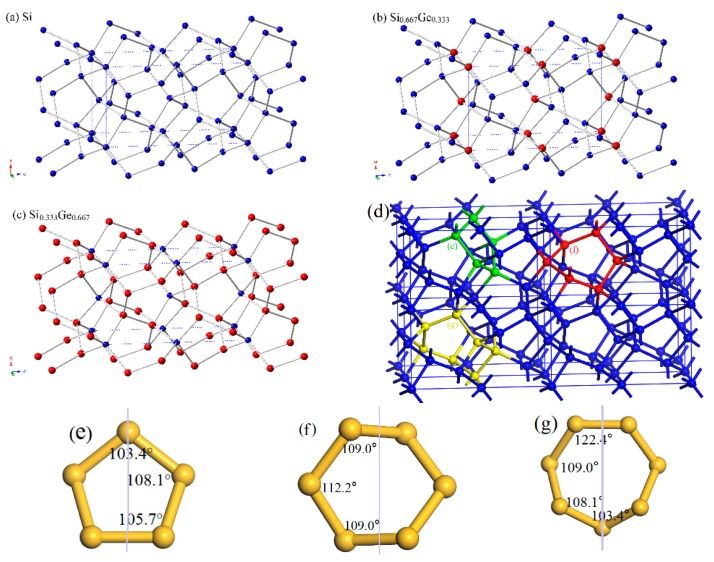
The crystal structures of Si (**a**), Si_0.667_Ge_0.333_ (**b**), Si_0.333_Ge_0.667_ (**c**) alloys in *P*4_2_/*ncm* phase. The blue and red spheres represent the Si atoms and Ge atoms. Si_5_, Si_6_ and Si_7_ rings in the 2 × 2 × 2 supercell (**d**). Si_5_ (**e**), Si_6_ (**f**) and Si_7_ (**g**) rings in the *P*4_2_/*ncm* phase.

**Figure 2:**
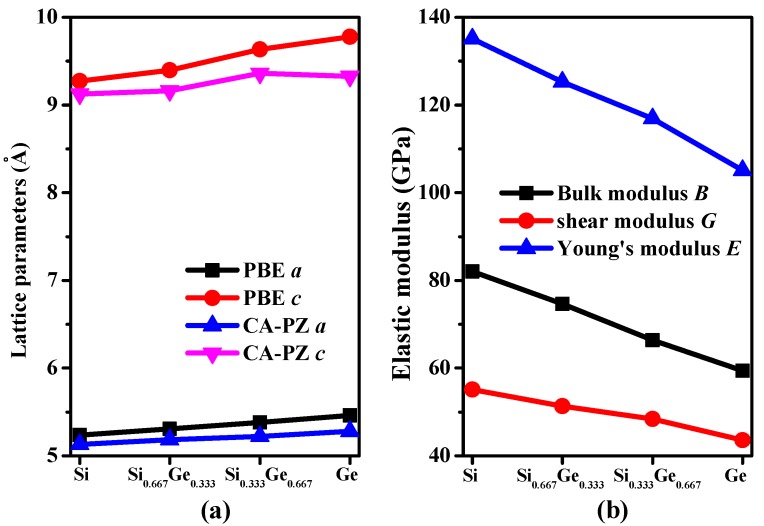
Lattice parameters (**a**) and elastic modulus (**b**) as a function of germanium concentration for Si–Ge alloys in *P*4_2_/*ncm* phase.

The crystal structures of Si_0.667_Ge_0.333_ and Si_0.333_Ge_0.667_ alloys, together with the Si (Ge) in *P*4_2_/*ncm* space group are displayed in Figure 1a–c. The structures of the silicon germanium alloys can be obtained with the lowest energy structure when the position of the silicon atom is replaced by the germanium atom. The optimal lattice parameters of Si (Ge), Si_0.667_Ge_0.333_, and Si_0.333_Ge_0.667_ alloys in *P*4_2_/*ncm* phase are listed in Table 1; in addition, the optimized lattice parameters and experiment values of diamond Si and diamond Ge are also listed in Table 1. From Table 1, it is clear that the lattice parameters of *P*4_2_/*ncm*-Si and *P*4_2_/*ncm*-Ge in this work are in excellent agreement with the previous report [19], and the optimized results of diamond Si and diamond Ge in this work are also in excellent agreement with experiment values [34,35]. At zero pressure, within *P*4_2_/*ncm* structure, two inequivalent atoms occupy the crystallographic 4*b* and 8*i* sites in the conventional cell, which are 4*b* Ge (0.0000, 1.0000, 0.5000), Si 8*i* (−0.1624, 0.3376, 0.3551) for Si_0.667_Ge_0.333_. For *P*4_2_/*ncm*-Si_0.333_Ge_0.667_, Si occupy the crystallographic 4*b* sites (0.0000, 1.0000, 0.5000), Ge occupy the crystallographic 4*b* sites (−0.1662, 0.3338, 0.3607). The energies of Si_0.667_Ge_0.333_ and Si_0.333_Ge_0.667_ alloys are the lowest when the silicon atoms and germanium atoms occupy these positions, respectively. The silicon atoms occupy the 4*b* sites and the silicon atoms occupy the 8*i* crystallographic sites consisting of five-membered, six-membered, and seven-membered silicon rings for *P*4_2_/*ncm*-Si. The position of five-membered, six-membered, and seven-membered silicon rings for *P*4_2_/*ncm*-Si are shown in Figure 1d. The angles of five-membered, six-membered, and seven-membered silicon rings are shown in Figure 1e–g. In this article, we used the six-membered silicon rings as an example. The distribution of angle for the six-membered silicon rings are in a symmetric fashion. From *P*4_2_/*ncm*-Si to *P*4_2_/*ncm*-Ge, with the composition of the Ge atom increasing, the change of the angle has no rule to be found. The top angle is 109.0° for *P*4_2_/*ncm*-Si, 108.6° for *P*4_2_/*ncm*-Si_0.667_Ge_0.333_, and it is changed to 110.0° for *P*4_2_/*ncm*-Si_0.333_Ge_0.667_, while the top angle is 109.5° for *P*4_2_/*ncm*-Ge. Just like the top angle, the middle angles are 112.2°, 111.2°, 112.4° and 111.4° for *P*4_2_/*ncm*-Si, *P*4_2_/*ncm*-Si_0.667_Ge_0.333_, *P*4_2_/*ncm*-Si_0.333_Ge_0.667_, and *P*4_2_/*ncm*-Ge, respectively. There is no law to follow, either. The lattice parameters of *P*4_2_/*ncm*-Si, *P*4_2_/*ncm*-Si_0.667_Ge_0.333_, *P*4_2_/*ncm*-Si_0.333_Ge_0.667_, and *P*4_2_/*ncm*-Ge as a function of the percentage of the Ge composition are shown in Figure 2a. From Figure 2a, it is obvious that the lattice parameters of Si-Ge alloys in *P*4_2_/*ncm* phase increases with the increasing percentage of the Ge composition. The larger the atomic radius of germanium is, the larger the chemical bond length between germanium and silicon atoms is. This is the reason why the lattice parameters increase.

### 3.2. Mechanical and Anisotropic Properties

The calculated elastic constants and elastic moduli of Si, Si_0.667_Ge_0.333_, Si_0.333_Ge_0.667_, Ge in *P*4_2_/*ncm* and *P*4_2_/*mnm* phases, together with diamond Si and diamond Ge are listed in Table 2. From Table 2, according to the mechanical stability criteria of tetragonal symmetry [36,37], the Si_0.667_Ge_0.333_ and Si_0.333_Ge_0.667_ alloys in the *P*4_2_/*ncm* phase both satisfy it. Since the *P*4_2_/*ncm* and *P*4_2_/*mnm* [18] phases both belong to the tetragonal symmetry, we mainly discussed the different mechanical properties between these two silicon allotropes. In addition, the elastic moduli of Si, Si_0.667_Ge_0.333_, Si_0.333_Ge_0.667_, Ge in *P*4_2_/*ncm* phase as a function of the percentage of the Ge composition are shown in Figure 2b. Obviously, the elastic moduli of *P*4_2_/*ncm*-Si are larger than that of *P*4_2_/*ncm*-Ge; in other words, the elastic moduli of Si-Ge alloys in *P*4_2_/*ncm* phase decreases with the increasing percentage of the Ge composition. The calculated elastic constants of *P*4_2_/*mnm* phase are almost smaller than those of P4_2_/*ncm* phase (see Table 2). In addition, Young’s modulus *E* and Poisson’s ratio *v* of *P*4_2_/*ncm* and *P*4_2_/*mnm* phases are also listed in Table 2. Young’s modulus and Poisson’s ratio are estimated by the following two formulas: *E* = 9*BG*/(3*B* + *G*), *v* = (3*B* − 2*G*)/(2(3*B* + *G*)). The calculated Young’s modulus of *P*4_2_/*ncm*-Si is 135 GPa, which is slightly smaller than that of diamond Si (155 GPa), but the Young’s modulus of *P*4_2_/*ncm*-Si is about two times than that of Si_96_ (69 GPa [8]) and slightly greater than that of *P*4_2_/*mnm* Si, while the calculated Young’s modulus of *P*4_2_/*ncm*-Ge is 105 GPa, which is also slightly smaller than that of diamond Ge (122 GPa) and slightly greater than that of *P*4_2_/*mnm* Ge (83 GPa [18]). Compared with *P*4_2_/*mnm* phase, the elastic constants and bulk modulus, shear modulus and Young’s modulus of Si_0.667_Ge_0.333_, Si_0.333_Ge_0.667_ alloys in the *P*4_2_/*ncm* phase are all slightly greater.

**Figure 3:**
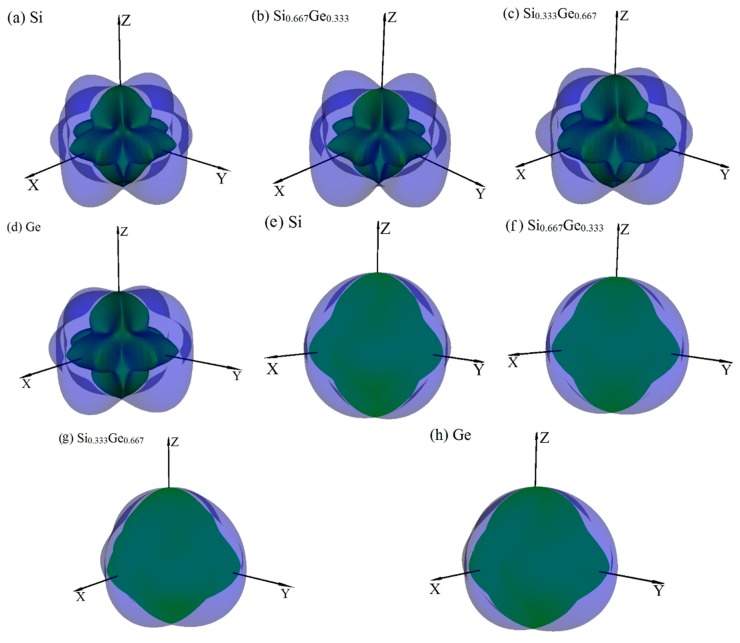
The surface constructions of Poisson’s ratio *v* (**a**–**d**) and shear modulus *G* (**e**–**h**) for Si, Ge and their Si_0.667_Ge_0.333_, Si_0.333_Ge_0.667_ alloys in the *P*4_2_/*ncm* phase. For all graphs, the units are in GPa.

**Figure 4:**
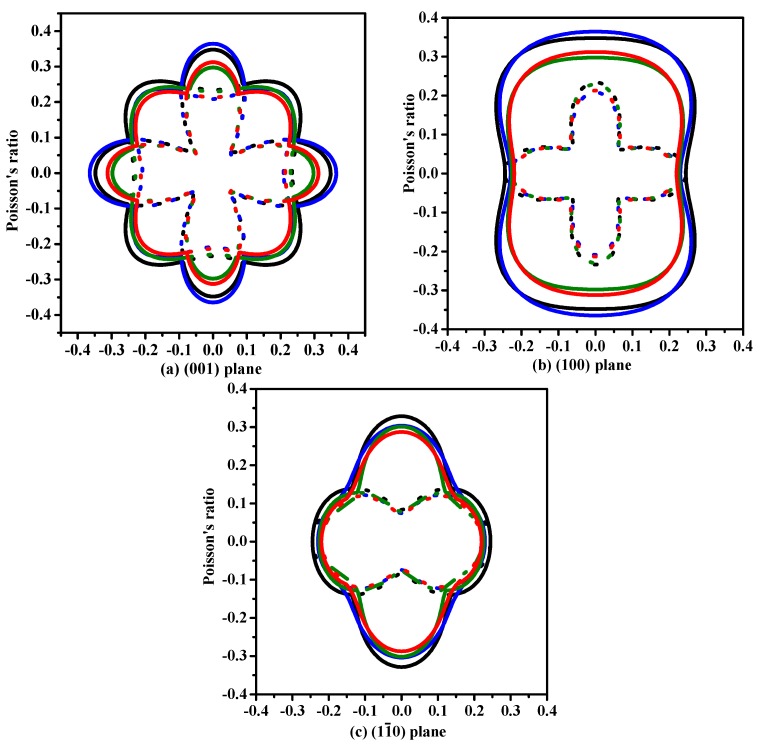
The two-dimensional representation of Poisson’s ratio in the (001) plane (**a**), (100) plane (**b**) and ($0\bar{1}0$) plane (**c**) for Si–Ge alloys in *P*4_2_/*ncm* structure.

The anisotropy is an important characteristic for materials. This work mainly discusses the mechanical anisotropy of Si, Ge and their Si_0.667_Ge_0.333_, Si_0.333_Ge_0.667_ alloys in the *P*4_2_/*ncm* phase. In our previous work [39], the methods we discussed are used to investigate the mechanical anisotropy. Based on the fundamental elastic constants, a useful visualization of the elastic anisotropy can be obtained by plotting a three-dimensional figure which shows the dependence of the Poisson’s ratio *v*, shear modulus *G*, and Young’s modulus *E* on a direction in a crystal. The three-dimensional surface representation of Poisson’s ratio *v* and shear modulus *G* for Si, Ge and their Si_0.667_Ge_0.333_, Si_0.333_Ge_0.667_ alloys in the *P*4_2_/*ncm* phase are illustrated in Figure 3a–h, respectively. The green and purple surface representation denotes the minimum and the maximum values of Poisson’s ratio *v* and shear modulus *G* along different directions, respectively. For an isotropic system, the three-dimensional directional dependence would exhibit a spherical shape, while the deviation degree from the spherical shape reflects the content of anisotropy [40]. From Figure 3a–h, it is clear that the shape of the three-dimensional directional dependence do not all exhibit a spherical shape, and the shape of the three-dimensional directional dependence for Si, Ge and their Si_0.667_Ge_0.333_, Si_0.333_Ge_0.667_ alloys in the *P*4_2_/*ncm* phase all exhibit mechanical anisotropy in Poisson’s ratio and shear modulus. Also, because the shape of the three-dimensional directional dependence of Si, Ge and their Si_0.667_Ge_0.333_, Si_0.333_Ge_0.667_ alloys in the *P*4_2_/*ncm* phase are not changed much, it follows that the mechanical properties in Poisson’s ratio and shear modulus should have little changes. The details of Poisson’s ratio (001), (100) and ($0\bar{1}0$) planes for Si, Ge and their Si_0.667_Ge_0.333_, Si_0.333_Ge_0.667_ alloys in the *P*4_2_/*ncm* phase are shown in Figure 4a–c, respectively. The black line, blue line, green line and red line represent the Poisson’s ratio of Si, Si_0.667_Ge_0.333_, Si_0.333_Ge_0.667_ and Ge in the *P*4_2_/*ncm* phase, respectively, and the solid line and dotted line denote the minimum and the maximum values of Poisson’s ratio *v* for Si, Si_0.667_Ge_0.333_, Si_0.333_Ge_0.667_ and Ge in the *P*4_2_/*ncm* phase, respectively. From Figure 4, the shape of the two-dimensional representation of Poisson’s ratio for Si, Si_0.667_Ge_0.333_, Si_0.333_Ge_0.667_ and Ge in the *P*4_2_/*ncm* phase has few changes. Moreover, for the same direction, the lines in different colors and types are very close to each other, which indicates that the Poisson’s ratio of Si, Si_0.667_Ge_0.333_, Si_0.333_Ge_0.667_ and Ge in *P*4_2_/*ncm* phase in this direction does not increase or decrease too much with the increasing percentage of the Ge composition. Under normal circumstances, the Poisson’s ratio should reduce with the increasing percentage of the Ge composition. While along *x*-axis ([100] direction), *y*-axis ([010] direction) and other unusual directions, the Poisson’s ratio of Si_0.667_Ge_0.333_ is slightly larger than that of Si in *P*4_2_/*ncm* phase; but in (1–10) plane, the Poisson’s ratio of Si_0.667_Ge_0.333_ in *P*4_2_/*ncm* phase along [001] direction is not larger than that of *P*4_2_/*ncm*-Si. The maximum value of Si, Si_0.667_Ge_0.333_ in *P*4_2_/*ncm* phase are both 0.37, and the Si_0.333_Ge_0.667_ and Ge in *P*4_2_/*ncm* phase are both 0.32, but they do not have the same location of the maximum values. The positions of the maximum values are located at *θ* = 1.00, *φ* = 0.00 (more details in the Refs. [39,41]); *θ* = 1.13, *φ* = 0.00; *θ* = 2.16, *φ* = 0.00 and *θ* = 1.08, *φ* = 0.00 for Si, Si_0.667_Ge_0.333_, Si_0.333_Ge_0.667_ and Ge in *P*4_2_/*ncm* phase, respectively; all angles are measured in radians. The maximum value of Si, Si_0.667_Ge_0.333_ Si_0.333_Ge_0.667_ and Ge in *P*4_2_/*ncm* phase all occupy the position *θ* = 1.57, *φ* = 3.93.

**Figure 5:**
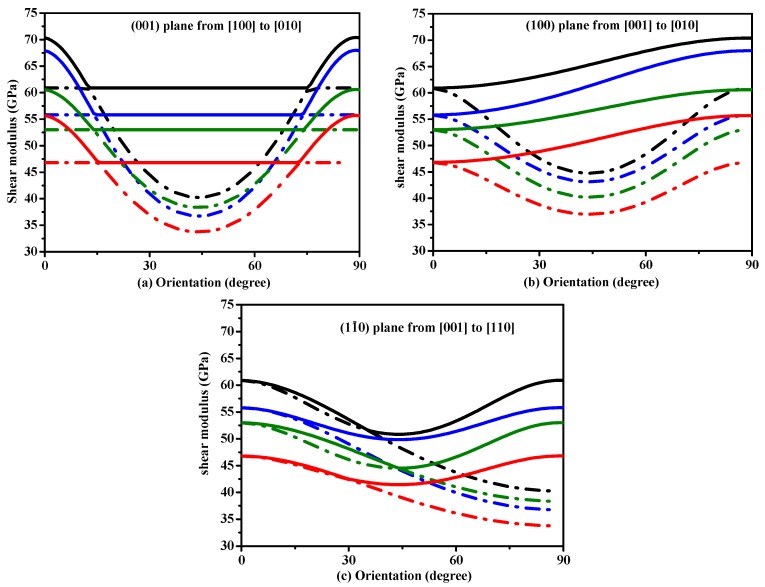
Orientation dependence of shear modulus in (001) plane (**a**), (100) plane (**b**) and ($0\bar{1}0$) plane (**c**) for Si, Si_0.667_Ge_0.333_, Si_0.333_Ge_0.667_ alloys and Ge in the *P*4_2_/*ncm* phase.

To understand plastic deformations in Si, Si_0.667_Ge_0.333_ Si_0.333_Ge_0.667_ and Ge in *P*4_2_/*ncm* phase, the variations of the shear modulus on stress direction are plotted in Figure 5. In Figure 5, in all the shear planes, the variation tendencies of shear moduli of Si, Si_0.667_Ge_0.333_ Si_0.333_Ge_0.667_ and Ge in *P*4_2_/*ncm* phase with the increasing percentage of the Ge composition are similar. In addition, the variation tendencies of shear moduli of Si, Si_0.667_Ge_0.333_ Si_0.333_Ge_0.667_ and Ge in *P*4_2_/*ncm* phase with the angle *γ* that is between the [100] and shear stress direction are also similar. For (001) shear plane, with the shear stress direction rotated from [100] direction to [010] direction, the shear moduli of *P*4_2_/*ncm*-Si are equal to each other when the angle *γ* is from 13° to 75°. The range of the same shear moduli of Si_0.667_Ge_0.333_ Si_0.333_Ge_0.667_ and Ge in *P*4_2_/*ncm* phase decreases with the increasing percentage of the Ge composition. Unlike (001) shear plane, with the shear stress direction rotated from [100] direction to [010] direction, there is no region with the same shear moduli in the (100) shear plane with the shear stress direction rotated from [001] direction to [010] direction and ($0\bar{1}0$) shear plane with the shear stress direction rotated from [001] direction to [110] direction. From Figure 5, the ordering of the shear modulus when the tensile axis is along a specific direction is: *G*_[100]_ ≈ *G*_[010]_ > *G*_[001]_ ≈ *G*_[110]_.

**Figure 6:**
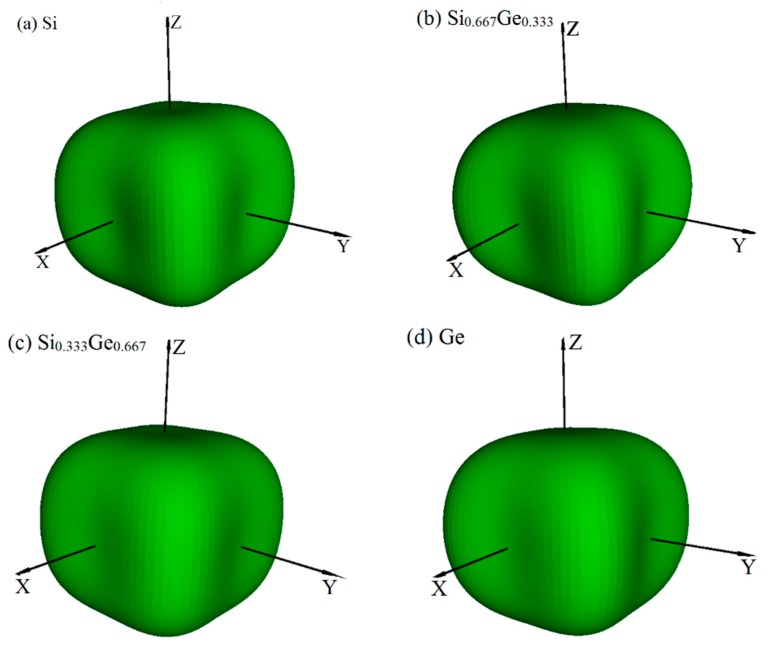
The surface constructions of Young’s modulus *E* for Si (**a**), Si_0.667_Ge_0.333_ (**b**), Si_0.333_Ge_0.667_ (**c**) and Ge (**d**) in the *P*4_2_/*ncm* phase. For all graphs, the units are in GPa.

**Figure 7:**
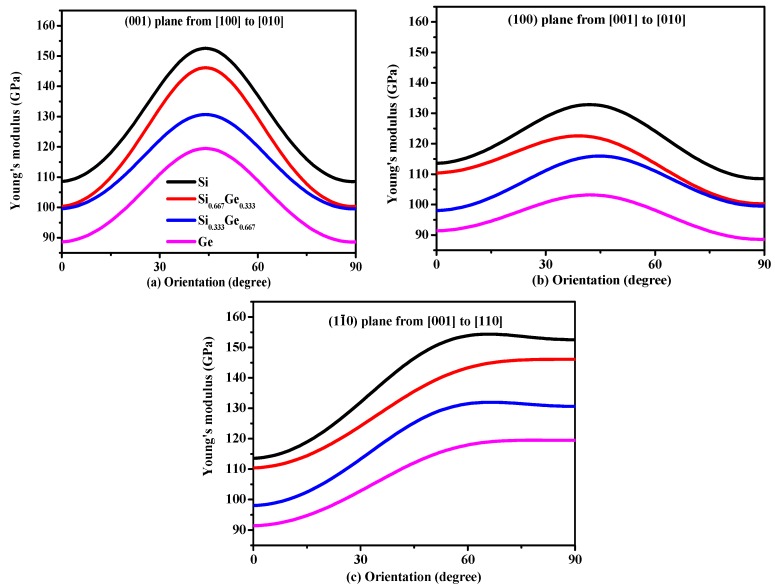
Orientation dependence of Young’s modulus in (001) plane (**a**), (100) plane (**b**) and ($0\bar{1}0$) plane (**c**) for Si, Si_0.667_Ge_0.333_, Si_0.333_Ge_0.667_ alloys and Ge in the *P*4_2_/*ncm* phase.

Young’s modulus is a measure of the stiffness of an isotropic elastic objective, which is a physical quantity that represents the nature of the material, and only depends on the physical properties of the material itself. The Young’s modulus indicates the rigidity of the material, and the bigger the Young’s modulus is, the less likely it is to deform. The three-dimensional surface representation of Young’s modulus for Si, Ge and their Si_0.667_Ge_0.333_, Si_0.333_Ge_0.667_ alloys in the *P*4_2_/*ncm* phase are illustrated in Figure 6a–d, respectively. Compared with Poisson’s ratio and shear modulus, Young’s modulus does not change too much with the increasing percentage of the Ge composition; Figure 6a–d are almost identical, except for the size of Young’s modulus. In addition, the Si, Si_0.667_Ge_0.333_, Si_0.333_Ge_0.667_ and Ge in *P*4_2_/*ncm* phase exhibit mechanical anisotropic properties in Young’s modulus. Figure 7 shows the variation of Young’s modulus in the (001) plane for the quadrant of directions [uvw] between [100] and [010], (100) plane for the quadrant of directions [uvw] between [001] and [010], and ($0\bar{1}0$) plane for the quadrant of directions [uvw] between [001] and [110], respectively. One can see that the largest Young’s modulus of *P*4_2_/*ncm*-Si_0.667_Ge_0.333_ is 146.1 GPa in (001) plane, and the *E*_[100]_ ≈ *E*_[010]_ = 100.3 GPa of Young’s modulus of *P*4_2_/*ncm*-Si_0.667_Ge_0.333_, the *E*_[100]_ ≈ *E*_[010]_ is also the smallest value of *P*4_2_/*ncm*-Si_0.667_Ge_0.333_ in (001) plane. In addition, *E*_[001]_ = 110.4 GPa in (100) plane for *P*4_2_/*ncm*-Si_0.667_Ge_0.333_, the smallest value of *P*4_2_/*ncm*- Si_0.667_Ge_0.333_ in (100) plane is 100.3 GPa in [010] direction. Also, in ($0\bar{1}0$) plane, the largest Young’s modulus of *P*4_2_/*ncm*-Si_0.667_Ge_0.333_ is 146.1 GPa, and the smallest value of *P*4_2_/*ncm*-Si_0.667_Ge_0.333_ in (1–10) plane is 110.4 GPa in [001] direction. It is worth noting that the Young’s modulus of *P*4_2_/*ncm*-Si_0.667_Ge_0.333_ and *P*4_2_/*ncm*-Ge increases with the increasing angle between the [100] and Young’s modulus stress direction, but for *P*4_2_/*ncm*-Si and *P*4_2_/*ncm* Si_0.333_Ge_0.667_, it increases first and then decreases with the increase of the angle. So, the (001) plane exhibits the largest mechanical anisotropic properties (in Young’s modulus) of the Si, Si_0.667_Ge_0.333_ Si_0.333_Ge_0.667_ and Ge in *P*4_2_/*ncm* phase, and the (100) plane exhibits the smallest mechanical anisotropic properties in Young’s modulus.

In addition, except for Poisson’s ratio, shear modulus and Young’s modulus, there is another physical quantity to characterize the mechanical anisotropy of a material: the universal anisotropic index *A*^U^ [42], which is defined as *A*^U^ = 5*G*_V_/*G*_R_ + *B*_V_/*B*_R_-6, where *G* and *B* represent shear modulus and bulk modulus, respectively, and the subscripts V and R denote the Voigt and Reuss approximations, respectively. The calculated universal anisotropic index of the Si, Si_0.667_Ge_0.333_, Si_0.333_Ge_0.667_ and Ge in *P*4_2_/*ncm* phase are also listed in Table 2. Compared with the Si, Si_0.667_Ge_0.333_, Si_0.333_Ge_0.667_ and Ge in *P*4_2_/*mnm* phase, the universal anisotropic index of the Si, Si_0.667_Ge_0.333_, Si_0.333_Ge_0.667_ and Ge in *P*4_2_/*ncm* phase is two times, or even almost three times that of the Si, Si_0.667_Ge_0.333_, Si_0.333_Ge_0.667_ and Ge in *P*4_2_/*mnm* phase, respectively. Among the Si, Si_0.667_Ge_0.333_, Si_0.333_Ge_0.667_ and Ge in *P*4_2_/*ncm* phase, *P*4_2_/*ncm*-Si exhibits the largest mechanical anisotropic properties in the universal anisotropic index than that of *P*4_2_/*ncm*-Ge, Si_0.667_Ge_0.333_ and Si_0.333_Ge_0.667_. Moreover, the universal anisotropic index of the *P*4_2_/*ncm*-Si is slightly larger than that of Si in *Amm*2, *C*2/*m*-16, *C*2/*m*-20 phases, much larger than that of Si in *I*-4 phase, while it is slightly smaller than that of diamond silicon (*Fd*-3*m* phase).

### 3.3. Electronic Properties

**Figure 8:**
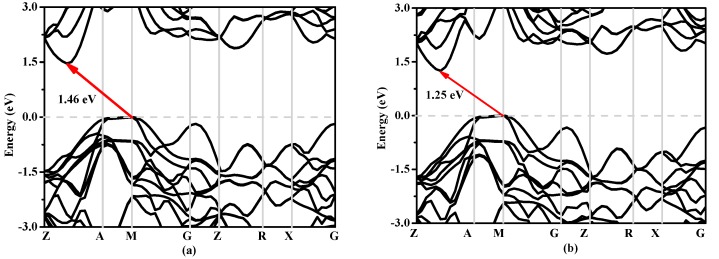

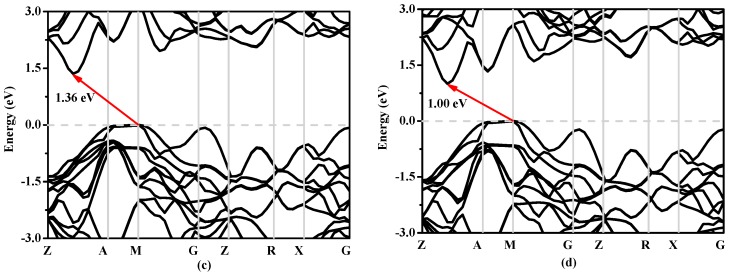
The band structures of Si (**a**), Si_0.667_Ge_0.333_ (**b**), Si_0.333_Ge_0.667_ (**c**) alloys and Ge (**d**) in the *P*4_2_/*ncm* phase.

In solid state physics, the energy band structure of a solid, also known as the electron band structure, describes the energy that is forbidden or permitted by electrons. The band structure of a material determines a variety of properties, especially its electronic and optical properties. The band structures of Si, Si_0.667_Ge_0.333_, Si_0.333_Ge_0.667_ and Ge in *P*4_2_/*ncm* phase calculated utilizing the Heyd–Scuseria–Ernzerhof (HSE06) [32,33] hybrid functional are shown in Figure 8a–d, respectively. The calculated band gaps of Si, Si_0.667_Ge_0.333_, Si_0.333_Ge_0.667_ and Ge in *P*4_2_/*ncm* phase are 0.63 eV, 0.58 eV, 0.55 eV and 0.39 eV utilizing the CA-PZ function, respectively; the band gaps of Si and Ge in *P*4_2_/*ncm* phase are in excellent agreement with the previous report [19]. In Figure 8, one can see that the band gaps of the Si, Si_0.667_Ge_0.333_, Si_0.333_Ge_0.667_ and Ge in *P*4_2_/*ncm* phase are all much larger than those of the CA-PZ function. From Figure 8, the *P*4_2_/*ncm*-Si, *P*4_2_/*ncm*-Ge and their alloys Si_0.667_Ge_0.333_, Si_0.333_Ge_0.667_ in *P*4_2_/*ncm* phase are all indirect band gaps with band gaps of 1.46 eV, 1.00 eV, 1.25 eV and 1.36 eV, respectively. The valence band maximum (VBM) of the *P*4_2_/*ncm*-Si, *P*4_2_/*ncm*-Ge and their alloys Si_0.667_Ge_0.333_, Si_0.333_Ge_0.667_ are all located at M point, while the conduction band maximum (VBM) of the *P*4_2_/*ncm*-Si, *P*4_2_/*ncm*-Ge and their alloys Si_0.667_Ge_0.333_, Si_0.333_Ge_0.667_ in *P*4_2_/*ncm* phase are all located at the point (0.196 0.196 0.5) along the Z-A direction. In addition, the Fermi level of Si, Ge and their alloys Si_0.667_Ge_0.333_, Si_0.333_Ge_0.667_ in *P*4_2_/*ncm* phase decreases with the increasing percentage of the Ge composition.

### 3.4. The Minimum Thermal Conductivity κ_min_

**Figure 9:**
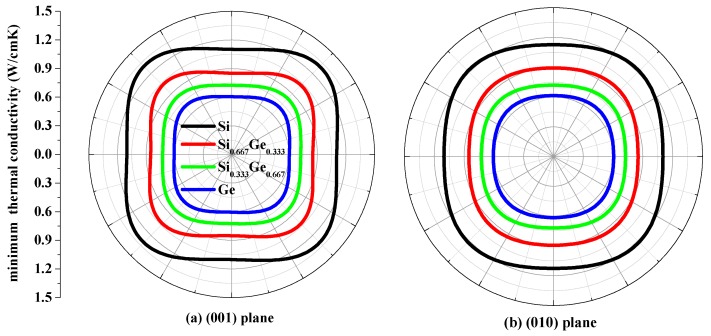

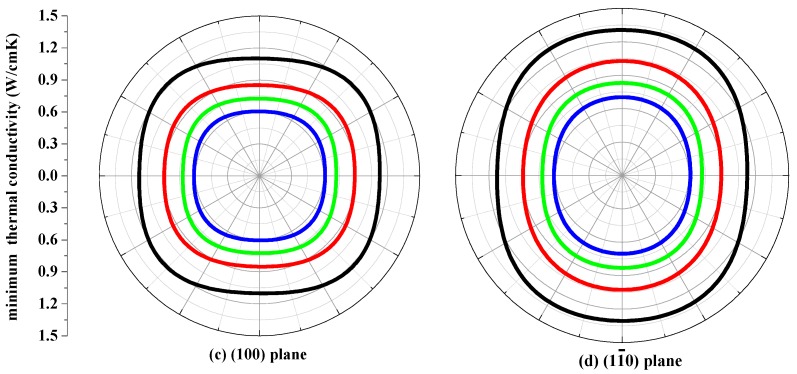
The minimum thermal conductivity κ_min_ of Si (**a**), Si_0.667_Ge_0.333_ (**b**), Si_0.333_Ge_0.667_ (**c**) alloys and Ge (**d**) in the *P*4_2_/*ncm* phase.

The thermal conductivity *κ* is the property of a material that conducts heat in physics. The minimum thermal conductivity *κ*_min_ can be calculated using the following theoretical method, namely, Clarke’s model [43], expressed as follows: *κ*_min_ = 0.87*k*_B_*m*^−2/3^(*E*/*ρ*)^1/2^, where *m* is the number of atoms in unit volume, and *m* = (*M*/*nρN*_A_), *M* is the molecular weight; *n* is the number of atoms in the unit cell, *k*_B_ is Boltzmann’s constant, *N*_A_ is Avogadro’s number, *ρ* is the density of the *P*4_2_/*ncm*-Si, *P*4_2_/*ncm*-Ge and their alloys Si_0.667_Ge_0.333_, Si_0.333_Ge_0.667_. The calculated minimum thermal conductivity *κ*_min_ of the *P*4_2_/*ncm*-Si, *P*4_2_/*ncm*-Ge and their alloys Si_0.667_Ge_0.333_, Si_0.333_Ge_0.667_ in different planes are plotted in Figure 9. The black, red, green and blue line representations denote the minimum thermal conductivity of the Si, Si_0.667_Ge_0.333_ Si_0.333_Ge_0.667_ and Ge in *P*4_2_/*ncm* phase, respectively. From Figure 9, the minimum thermal conductivity of the Si, Si_0.667_Ge_0.333_, Si_0.333_Ge_0.667_ and Ge in *P*4_2_/*ncm* phase exhibits varying degrees of anisotropic properties in different planes. In (001), (010) and (100) planes, the values of the minimum thermal conductivity *κ*_min_ is almost a square with four rounded vertex angles, while the minimum thermal conductivity *κ*_min_ in ($0\bar{1}0$) plane is like an inverted ellipse. The minimum thermal conductivity *κ*_min_ of the Si, Si_0.667_Ge_0.333_, Si_0.333_Ge_0.667_ and Ge in *P*4_2_/*ncm* phase decreases with the increasing percentage of the Ge composition, as will be readily seen. Also, the minimum thermal conductivity *κ*_min_ decreases more and more slowly with the increasing percentage of the Ge composition.

## 4. Conclusions

In summary, we have predicted the structural, mechanical, anisotropic and thermal properties of two silicon germanium alloys with space group *P*4_2_/*ncm* using the density functional theory. The calculated lattice parameters of the *P*4_2_/*ncm*-Si, *P*4_2_/*ncm*-Ge are in excellent agreement with the previous report. The elastic constants and elastic moduli of the Si, Si_0.667_Ge_0.333_, Si_0.333_Ge_0.667_ and Ge in *P*4_2_/*ncm* phase are calculated using strain-stress method. Compared with *P*4_2_/*mnm* phase, the elastic constants and bulk modulus, shear modulus and Young’s modulus of Si_0.667_Ge_0.333_, Si_0.333_Ge_0.667_ alloys in *P*4_2_/*ncm* phase are all slightly greater. The Si, Si_0.667_Ge_0.333_, Si_0.333_Ge_0.667_ and Ge in *P*4_2_/*ncm* phase exhibit different degrees of mechanical anisotropic properties in Poisson’s ratio, shear modulus, Young’s modulus, and universal anisotropic index. From the band structures of Si, Si_0.667_Ge_0.333_, Si_0.333_Ge_0.667_ and Ge in *P*4_2_/*ncm* phase, we can conclude that the Si, Si_0.667_Ge_0.333_, Si_0.333_Ge_0.667_ and Ge in *P*4_2_/*ncm* phase are all indirect band gap semiconductors. By adjusting the composition of germanium atoms, Si, Si_0.667_Ge_0.333_, Si_0.333_Ge_0.667_ and Ge in *P*4_2_/*ncm* phase can be used to make different semiconductor devices. In addition, the minimum thermal conductivity *κ*_min_ of Si, Si_0.667_Ge_0.333_, Si_0.333_Ge_0.667_ and Ge in *P*4_2_/*ncm* phase in different planes are also investigated in this work; the results show that the minimum thermal conductivity *κ*_min_ of the Si, Si_0.667_Ge_0.333_, Si_0.333_Ge_0.667_ and Ge in *P*4_2_/*ncm* phase exhibit different degrees of anisotropic properties, and the thermal conductivity *κ*_min_ of the Si, Si_0.667_Ge_0.333_, Si_0.333_Ge_0.667_ and Ge in *P*4_2_/*ncm* phase has the largest anisotropy properties in ($0\bar{1}0$) plane.

## Figures and Tables

**Table 1:** The lattice parameters (in Å) of Si, Si_0.667_Ge_0.333_, Si_0.333_Ge_0.667_ and Ge alloys in *P*4_2_/*ncm* phase.

Materials	PBE		CA-PZ		Experimental
	*a*	*c*	*a*	*c*	*a*
Si	5.237	9.274	5.132	9.125	-
	-	-	5.135 ^1^	9.167	-
Si_0.667_Ge_0.333_	5.306	9.397	5.185	9.163	-
Si_333_Ge_0.667_	5.380	9.635	5.222	9.361	-
Ge	5.462	9.779	5.281	9.396	-
	-	-	5.292 ^1^	9.346	-
Diamond-Si	5.441	-	5.419	-	5.431 ^2^
Diamond-Ge	5.699	-	5.580	-	5.660 ^2^

^1^ Ref [19], ^2^ Ref [34,35].

**Table 2:** The calculated elastic constants (in GPa) and elastic modulus (in GPa) of Si_0.667_Ge_0.333_, Si_0.333_Ge_0.667_ in *P*4_2_/*ncm* and *P*4_2_/*mnm* phases and Si, Ge in *P*4_2_/*ncm*, *P*4_2_/*mnm* phases and diamond phases.

Materials	*C*_11_	*C*_12_	*C*_13_	*C*_33_	*C*_44_	*C*_66_	*B*	*G*	*E*	*v*	*A*^U^
*P*4_2_/*ncm*-Si	141	61	49	138	61	73	82	55	135	0.226	0.237
Si_0.667_Ge_0.333_	130	56	43	130	56	68	75	51	125	0.220	0.225
Si_0.333_Ge_0.667_	122	45	38	115	53	61	66	48	117	0.207	0.156
*P*4_2_/*ncm*-Ge	108	41	33	106	47	56	59	44	105	0.205	0.158
*P*4_2_/*mnm*-Si ^1^	123	47	48	146	49	61	75	48	119	0.236	0.134
Si_0.667_Ge_0.333_^1^	111	36	42	130	43	38	66	40	100	0.252	0.035
Si_0.333_Ge_0.667_^1^	100	32	37	110	39	44	58	38	94	0.231	0.056
*P*4_2_/*mnm*-Ge ^1^	88	26	32	100	35	40	50	34	83	0.223	0.054
Diamond-Si	160	62	-	-	79	-	95	65	159	0.221	0.279
	166 ^2^	64	-	-	80	-	102	-	-	-	-
Diamond-Ge	123	47	-	-	62	-	72	51	124	0.213	0.293
	129 ^2^	48	-	-	67	-	77	-	-	-	-

^1^ Ref [18], ^2^ Ref [38]-experiment.

## References

[1] Wu F., Jun D., Kan E.J., Li Z.Y. (2011). Density functional predictions of new silicon allotropes: Electronic properties and potential applications to Li-battery anode materials. Solid State Commun..

[2] Fujimoto Y., Koretsune T., Saito S., Miyake T., Oshiyama A. (2008). A new crystalline phase of four-fold coordinated silicon and germanium. New J. Phys..

[3] De A., Pryor C.E. (2014). Electronic structure and optical properties of Si, Ge and diamond in the lonsdaleite phase. J. Phys. Condens. Matter.

[4] Malone B.D., Sau J.D., Cohen M.L. (2008). Ab initio survey of the electronic structure of tetrahedrally bonded phases of silicon. Phys. Rev. B.

[5] Fan Q.Y., Chai C.C., Wei Q., Yan H.Y., Zhao Y.B., Yang Y.T., Yu X.H., Liu Y., Xing M.J., Zhang J.Q. (2015). Novel silicon allotropes: Stability, mechanical, and electronic properties. J. Appl. Phys..

[6] Xiang H.J., Huang B., Kan E.J., Wei S.H., Gong X.G. (2013). Towards direct-gap silicon phases by the inverse band structure design approach. Phys. Rev. Lett..

[7] Wang Q.Q., Xu B., Sun J., Liu H.Y., Zhao Z.S., Yu D.L., Fan C.Z., He J.L. (2014). Direct band gap silicon allotropes. J. Am. Chem. Soc..

[8] He C.Y., Zhang C.X., Li J., Peng X.Y., Meng L.J., Tang C., Zhong J.X. (2016). Direct and quasi-direct band gap silicon allotropes with remarkable stability. Phys. Chem. Chem. Phys..

[9] Amsler M., Flores-Livas J.A., Lehtovaara L., Balima F., Ghasemi S.A., Machon D., Pailhes S., Willand A., Caliste D., Botti S. (2012). Crystal Structure of Cold Compressed Graphite. Phys. Rev. Lett..

[10] Pfrommer B.G., Cote M., Louie S.G., Cohen M.L. (1997). Ab initio study of silicon in the R8 phase. Phys. Rev. B.

[11] Fan Q.Y., Chai C.C., Wei Q., Yang Y.T., Yang Q., Chen P.Y., Xing M.J., Zhang J.Q., Yao R.H. (2016). Prediction of novel phase of silicon and Si–Ge alloys. J. Solid State Chem..

[12] Zhang X.D., Ying C.H., Quan S.Y., Shi G.M., Li Z.J. (2012). A first principles investigation on the structural, phonon, elastic and thermodynamic properties of the Si0.5Sn0.5 cubic alloy. Solid State Commun..

[13] Zhang Y.X., Xiang G., Gu G.X., Li R., He D.W., Zhang X. (2012). Nonlinear concentration-dependent electronic and optical properties of Si_1–*x*_Ge*_x_* alloy nanowires. J. Phys. Chem. C.

[14] Zhang X.D., Ying C.H., Li Z.J., Shi G.M. (2012). First-principles calculations of structural stability, elastic, dynamical and thermodynamic properties of SiGe, SiSn, GeSn. Superlattices Microstruct..

[15] Zhu Y., Zhang X.Y., Zhang S.H., Sun X.W., Wang L.M., Ma M.Z., Liu R.P. (2014). First-principles investigations on thermodynamic properties of the ordered and disordered Si0.5Ge0.5 alloys. Appl. Phys. A.

[16] Bautista-Hernandez A., Rangel T., Romero A.H., Rignanese G.M., Salazar-Villanueva M., Chigo-Anota E. (2013). Structural and vibrational stability of M and Z phases of silicon and germanium from first principles. J. Appl. Phys..

[17] Zhang Y.H., Chai C.C., Fan Q.Y., Yang Y.T., Xing M.J. (2016). Mechanical and electronic properties of Si-Ge alloy in Cmmm structure. Chin. J. Phys..

[18] Fan Q.Y., Chai C.C., Wei Q., Yang Q., Zhou P.K., Xing M.J., Yang Y.T. (2016). Mechanical and electronic properties of Si, Ge and their alloys in *P*4_2_/*mnm* structure. Mater. Sci. Semicond. Process..

[19] Zhao Z.S., Tian F., Dong X., Li Q., Wang Q.Q., Wang H., Zhong X., Xu B., Yu D., He J.L. (2012). Tetragonal allotrope of group 14 Elements. J. Am. Chem. Soc..

[20] Hohenberg P., Kohn W. (1964). Inhomogeneous electron gas. Phys. Rev..

[21] Kohn W., Sham L.J. (1965). Self-consistent equations including exchange and correlation effects. Phys. Rev..

[22] Clark S.J., Segall M.D., Pickard C.J., Hasnip P.J., Probert M.I.J., Refson K., Payne M.C. (2005). First principles methods using CASTEP. Z. Kristallogr..

[23] Monkhorst H.J., Pack J.D. (1976). Special points for Brillouin-zone integrations. Phys. Rev. B.

[24] Vanderbilt D. (1990). Soft self-consistent pseudopotentials in a generalized eigenvalue formalism. Phys. Rev. B.

[25] Perdew J.P., Zunger A. (1981). Self-interaction correction to density-functional approximations for many-electron systems. Phys. Rev. B.

[26] Ceperley D.M., Alder B.J. (1980). Ground State of the Electron Gas by a Stochastic Method. Phys. Rev. Lett..

[27] Perdew J.P., Burke K., Ernzerhof M. (1996). Generalized gradient approximation made simple. Phys. Rev. Lett..

[28] Pfrommer B.G., Côté M., Louie S.G., Cohen M.L. (1997). Relaxation of crystals with the quasi-newton method. J. Comput. Phys..

[29] Baroni S., de Gironcoli S., dal Corso A., Giannozzi P. (2001). Phonons and related crystal properties from density-functional perturbation theory. Rev. Mod. Phys..

[30] Gueorguiev G.K., Pacheco J.M. (2003). Shapes of cagelike metal carbide clusters: First-principles calculations. Phys. Rev. B.

[31] Gueorguiev G.K., Broitman E., Furlan A., Stafström S., Hultman L. (2009). Dangling bond energetics in carbon nitride and phosphorus carbide thin films with fullerene-like and amorphous structure. Chem. Phys. Lett..

[32] Krukau A.V., Vydrov O.A., Izmaylov A.F., Scuseria G.E. (2006). Influence of the exchange screening parameter on the performance of screened hybrid functionals. J. Chem. Phys..

[33] Fan Q.Y., Chai C.C., Wei Q., Yang Y.T. (2016). Two novel silicon phases with direct band gaps. Phys. Chem. Chem. Phys..

[34] Lide D.R. (1992). CRC Handbook of Chemistry and Physics.

[35] Cohen E.R., Taylor B.N. (1987). The 1986 adjustment of the fundamental physical constants. Rev. Mod. Phys..

[36] Wu Z.J., Zhao E.J., Xiang H.P., Hao X.F., Liu X.J., Meng J. (2007). Crystal structures and elastic properties of superhard IrN_2_ and IrN_3_ from first principles. Phys. Rev. B.

[37] Fan Q.Y., Chai C.C., Wei Q., Yang Y.T. (2016). Two novel C_3_N_4_ phases: Structural, mechanical and electronic properties. Materials.

[38] Gomez-Abal R., Li X.Z., Scheffler M., Ambrosch-Draxl C. (2008). Influence of the core-valence interaction and of the pseudopotential approximation on the electron self-energy in semiconductors. Phys. Rev. Lett..

[39] Ma Z.Y., Han Z., Liu X.H., Yu X.H., Wang D.Y., Tian Y. (2017). Pnma-BN: Another Boron Nitride polymorph with interesting physical properties. Nanomaterials.

[40] Hu W.C., Liu Y., Li D.J., Zeng X.Q., Xu C.S. (2014). First-principles study of structural and electronic properties of C14-type Laves phase Al_2_Zr and Al_2_Hf. Comput. Mater. Sci..

[41] Marmier A., Lethbridge Z.A.D., Walton R.I., Smith C.W., Parker S.C., Evans K.E. (2010). ElAM: A computer program for the analysis and representation of anisotropic elastic properties. Comput. Phys. Commun..

[42] Ranganathan S.I., Ostoja-Starzewski M. (2008). Universal elastic anisotropy index. Phys. Rev. Lett..

[43] Long J.P., Shu C.Z., Yang L.J., Yang M. (2015). Predicting crystal structures and physical properties of novel superhard p-BN under pressure via first-principles investigation. J. Alloys Compd..

